# Further Evidence of the Zero-Association Between Symptoms of Insomnia and Facial Emotion Recognition—Results From a Sample of Adults in Their Late 30s

**DOI:** 10.3389/fpsyt.2018.00754

**Published:** 2019-01-17

**Authors:** Serge Brand, René Schilling, Sebastian Ludyga, Flora Colledge, Dena Sadeghi Bahmani, Edith Holsboer-Trachsler, Uwe Pühse, Markus Gerber

**Affiliations:** ^1^Division of Sport Science and Psychosocial Health, Department of Sport, Exercise and Health, University of Basel, Basel, Switzerland; ^2^Center of Affective, Stress and Sleep Disorders, Psychiatric Clinics (UPK), University of Basel, Basel, Switzerland; ^3^Substance Abuse Prevention Research Center, Kermanshah University of Medical Sciences, Kermanshah, Iran; ^4^Sleep Disturbances Research Center, Kermanshah University of Medical Sciences, Kermanshah, Iran; ^5^Isfahan Neurosciences Research Center, Alzahra Research Institute, Isfahan University of Medical Sciences, Isfahan, Iran

**Keywords:** symptoms of insomnia, facial emotion recognition, late young adulthood, mental toughness, perceived stress

## Abstract

**Background:** Restoring sleep is associated with favorable cognitive, emotional, and behavioral adaptations. As regards the association between sleep duration and facial emotion recognition (FER), results are conflicting, and as regards the association between symptoms of insomnia and FER, no study has been performed so far. Accordingly, the aim of the present study was to investigate whether subjective sleep was associated with FER, along with perceived stress and mental toughness.

**Method:** A total of 201 police officers (mean age = 38.5 years, 64.2% males) took part in the present cross-sectional study. They completed questionnaires covering socio-demographic data, subjective symptoms of insomnia, perceived stress, and mental toughness. Further, they underwent a computerized FER test, consisting of facial emotion labeling and facial emotion matching.

**Results:** Performance of FER (accuracy, speed) was unrelated to subjective symptoms of insomnia. Lower FER was associated with higher age, but not to perceived stress or mental toughness. No gender differences were observed. Higher symptoms of insomnia were associated with higher stress scores and lower scores of mental toughness.

**Conclusions:** The pattern of results suggests that FER was not associated with symptoms of insomnia, understood as a proxy of sleep quality, among adults. This observation replicates those studies showing a zero-association between sleep and FER.

## Introduction

The human brain has evolved and grown to its current dimensions and structure to cope with complex social environments (1, 2). Specifically, the prefrontal cortex has evolved during the last 1.8 mio years as a result of humans living in groups consisting of 8–12 families and up to 120–150 individuals with increasingly complex interactions and information demands (3). In this view and in line with Social Contract Theory, Cosmides et al. (3) suggest that humans dispose of five unique core abilities, namely, the ability to (1) recognize many different individual people, to (2) remember the histories of interactions with these individuals, to (3) communicate one's values to others, to (4) understand other individuals' values, and to (5) understand costs and benefits of interactions and values of items. Furthermore, throughout history, individuals often had to identify possible fair-weather friends (“friends” who stay committed as long as we do not need real help and who abandon us when we are most in need of support), cheaters (individuals breaking rules of social contracts), free-riders (individuals benefiting from the group's performance without incurring personal costs), and defectors (individuals who benefit from the group's performance, but leave the group as soon as they should increase their personal costs). Not surprisingly, organisms such as mammals need time to acquire knowledge on social complexity and to learn how to cope with social challenges; accordingly, adolescence constitutes a relatively long period in human mankind, lasting much longer compared to all other mammals and animals (4).

A crucial dimension of social interaction is the accurate perception and interpretation of human faces (5). The human face expresses the individual's current emotional state, and correctly identifying an individual's current emotional state is crucial for one's security (6–8). Consequently, specific brain areas have evolved to identify faces and their underlying emotional processes (9). Damage to these areas leads to prosopagnosia, a specific neurocognitive impairment in perceiving and understanding human faces and their underlying emotional processes (10–12). This is problematic, as the Social Contract Theory (3) claims, among other things, that identifying and remembering faces of individuals breaking social rules is crucial for coping with cheaters, free-riders, or defectors.

Moreover, functional deficits in emotion processing and the respective response selection have been associated with poor social functioning (13), and both internalizing and externalizing problems among children and adolescents (14, 15). Additionally, impairments in emotion processing have been associated with difficulties in building up relationships, resulting in rejection by peers and/or in solitary play in children (16). Further, Ding et al. (17) underlined that perceived chronic social adversities may lead to symptoms of stress, along with depression and anxiety. In this regard, social interactions are tightly related to emotion regulation. To illustrate, Visted et al. (18) have shown in their systematic review and meta-analysis that people with a history of depressive episodes (current or remitted state) reported more difficulties with emotion regulation than never-depressed controls. Strikingly, such difficulties in emotion regulation appear to persist, even if symptoms of depression decrease. Accordingly, Visted et al. (18) suggested that poor emotion regulation and lower social competencies may be a latent risk factor for relapses. To summarize, evolving accurate emotion recognition abilities such as FER appears to be crucial for healthy psychosocial development.

A neurophysiological process to enable an optimal psychological functioning is sleep. Specifically, sufficient and restoring sleep is associated with a broad range of healthy cognitive (19–23), behavioral (24–26), and emotional processes (20, 27–36). With regard to emotion processing and affect, Bouwmans et al. (37) showed that sleep quality impacted on affect and mood the following day.

By contrast, a mixed overall pattern appears regarding the association between sleep and facial emotion recognition (FER), with some studies showing that shortened sleep duration was associated with poorer FER (38–42), while others did not or not fully (43–46) support such a relationship. In two recent reviews, de Almondes et al. (9) found 10 studies on this topic, and Holding et al. (44) identified two further investigations (43, 47). A deeper inspection of these 12 studies points toward a broad heterogeneity in study populations, sample size, methodological approaches to assess FER, along with manipulation of sleep duration ranging from no change to 72 h of sleep deprivation.

The present study will address several shortcomings identified in previous research. With regard to the population under study, most investigations focused on young healthy adults (38, 40–43, 46, 48–51), whereas only two studies assessed clinical samples (45, 47). Kyle et al. (45) found that compared to healthy controls (*N* = 15), patients with insomnia (*N* = 16; mean age of the total sample of 31 participants: 47.1 years, 66% females) showed a decrease in perceived intensity of sad and fearful faces, while no differences were observed as regards the categorization of FER. Moreover, Chu et al. (47) showed in a sample of 30 patients (mean age: 39 years) with severe schizophrenia disorders that improvements in sleep were associated with a more accurate recognition of the six basic emotions (happiness, fear, anger, disgust, sadness, surprise). While we did not assess a clinical population in our study, our intention was to examine the association between sleep and emotion regulation in a broader sample of adults. In the present study, we focused on police officers because this professional group might be exposed to stressful circumstances at work such as challenging social interactions (52, 53).

Additionally, previous studies have often been performed with small samples with <100 participants (38–43, 48–51), which poses the risk of underpowered statistical analyses. Currently, we know of only two studies composed of more than 100 participants (44, 46). Moreover, several studies employed the paradigm of sleep deprivation, ranging from one night of shortened sleep [4 h; (40); 8 h, (51)], to a full night of sleep deprivation (38, 39, 42, 44, 50), to 36 h (43), 47 h (49), or even 72 h (41). Only three studies assessed sleep under naturalistic conditions (44, 45, 47). Previous research has also shown that the associations between impaired or shortened sleep duration and the performance of FER are not robust (9, 44): To illustrate, Ginani et al. (43) found no association between total sleep deprivation for 36 h and FER of the five out of six basic emotions (exception: disgust). Maccari et al. (50) found no impact of a one night sleep deprivation on FER, compared to emotional words. Maccari et al. (50) concluded that positive emotions appeared to be even more resistant to sleep loss. In the study by van der Helm et al. (42), total sleep deprivation had an impact on FER of angry and happy faces at moderate, but not extreme intensity, a pattern of results observed exclusively among female, but not male participants. Likewise, Huck et al. (49) found no differences in the accuracy to discriminate and label simple affective faces (while there were differences in the accuracy to discriminate and label complex affective faces). In Cote et al. (38), a total sleep deprivation led to a less accurate categorization of sad, but not of happy, angry, or fearful faces. In Motomura et al. (40), a shortened sleep duration (4 h) led to a faster subliminal reactivity to fearful faces. In a similar way, Pallesen et al. (41) found a deterioration of speed and accuracy of FER after 72 h of sleep deprivation. Finally, only one study (44) focused on the relationship between FER and sleep disturbances (beyond the impact on sleep duration). The lack of research with regard to symptoms of insomnia are surprising as there is evidence that sleep quality, but not sleep quantity, is associated with a broad range of psychological processes (54–56).

Given this background, the present study expands previous research in several ways. First, we assessed a larger sample of 201 participants. Instead of healthy young students, we focused on a broader sample of police officers composed of people of different age groups, men and women, part- and full-time employees, shift and non-shift workers, and different professional hierarchical levels. Second, we focused on subjective symptoms of insomnia as a proxy of sleep quality, but not sleep duration. Likewise, participants were not artificially sleep-deprived; rather we focused on sleep problems occurring during naturalistic circumstances. Third, we employed a FER paradigm, where stimuli were presented at about 250 ms, thus assessing FER and facial emotion labeling at subliminal level. This approach entails that stimuli are perceived and elaborated at a pre-attentive level. Fourth, age, gender, perceived stress and mental toughness were assessed and introduced as further confounders. The inclusion of the latter two variables was deemed important as previous studies showed that poor sleep quality was associated with increased perceived stress (57) and lower scores of mental toughness (58, 59).

Taken together, and given the inconclusive nature of the literature and the research gaps highlighted above, we purposely decided against formulating clear-cut hypotheses on how subjective symptoms of insomnia might be associated with facial emotional recognition in a broader sample of adults. Further, following van der Helm et al. (42), we expected that FER would be more impaired in females, compared to males.

## Methods

### Procedure

Employees of the Police Force of the Canton of Basel-City (Basel, Switzerland) were approached to participate in a cross-sectional and longitudinal study on physical and mental health. They were fully informed about the aims of the study, the voluntary basis of participation and anonymous data handling. Thereafter, all participants signed the written informed consent, and completed self-rating questionnaires covering socio-demographic information, sleep disturbances, perceived stress, and mental toughness. Further, they completed a computerized test of FER (see below). Data from this ongoing study have not been published so far; accordingly, all data are novel. The local ethics committee (Ethikkommission Nordwest- und Zentralschweiz, Basel, Switzerland) approved the entire study (approval number−2017-01477), which was performed in accordance with the rules laid down at the Declaration of Helsinki and its later amendments.

### Sample

A total of ~1,000 employees of the Police Force of the Canton of Basel-City were eligible for the study; of those, 201 (~20%) agreed to participate in the current study. Mean age was 38.55 years (64.2% males). Descriptively, female participants were younger (*M* = 36.86 years; SD = 9.22), compared to their male counterparts (*M* = 39.50 years; SD = 10.52; *t*_(199)_ = 1.78; *p* = 0.07; *d* = 0.27). Inclusion criteria were: (1) All questions ticked “No” on an extended version of the Physical Activity Readiness Questionnaire; (2) No current illness; (3) Normal or corrected-to-normal vision.

The educational background was as follows: Vocational school (*n* = 4); high school degree (*n* = 158); university degree (*n* = 25); no answer (*n* = 14).

Shift cycles were the same for all shift workers. Each cycle consisted of 6 days. Day 1 was a day shift from 7 a.m. to 7 p.m. Day 2 was a late shift starting at 12 a.m. to 9 p.m. Day 3 was an early shift from 7 a.m. to 12 a.m., followed by a night shift starting at 7 p.m. to 7 a.m. the fourth day. The fifth and sixth days were off. All assessments took place in the afternoons from day 1 or day 2.

### Tools

#### Sociodemographic Data

Participants completed a questionnaire on age, and gender.

#### Subjective Symptoms of Insomnia

To assess subjective symptoms of insomnia, as in previous studies (28, 60–64), the Insomnia Severity Index [ISI; (65, 66)] was employed. Briefly, the questionnaire is a seven-item screening measure for insomnia and an outcome measure for use in treatment research. The items, answered on 5-point rating scales ranging from 0 (not at all) to 4 (very much), refer in part to the criteria for insomnia by assessing difficulty in falling asleep, difficulties remaining asleep, early morning awakenings, impaired daytime performance, low satisfaction with sleep, and worry about sleep. The higher the overall score, the more the participant is assumed to suffer from insomnia (Cronbach's alpha = 0.84). The ISI proved to be a valid and reliable measure to assess sleep disturbances in previous studies (66).

#### Mental Toughness

To assess mental toughness, as in previous studies (58, 59, 63, 67, 68) the short form (MTQ18) of the Mental Toughness Questionnaire 48 (69) was employed. It consists of 18 items focusing on commitment, confidence, control, and challenge. Answers are given on 5-point Likert scales from 1 (= strongly disagree) to 5 (= strongly agree), with higher scores reflecting higher mental toughness (Cronbach's alpha = 0.79). Evidence for the validity of the MTQ has been provided both for the 48-item long-form (70, 71) and the 18-item short form (72).

#### Perceived Stress

To assess perceived stress over the previous month, as in previous studies (73, 74), the Perceived Stress Scale (75) was employed. It consists of 10 items, and answers are given on five-point rating scales ranging from 1 (= never) to 5 (= very often), with higher scores reflecting greater perceived stress (Cronbach's alpha = 0.74).

#### Facial Emotion Recognition

The assessment of FER was performed with one person at a time. Facial emotion recognition was measured with two computerized tests, namely emotion labeling and emotion matching. The tasks were administered with E-Prime 2.0 (Psychology Software Tools, USA). We used static emotions. The surrounding noise was kept to a minimum and the environmental temperature was held constant.

Prior to testing, instructions were given verbally and presented on the screen to make sure participants understood the task. During emotion labeling, a target face was presented centrally and participants were required to choose the appropriate emotion from a pair of emotions presented at the bottom of the screen. During emotion matching, the emotions were replaced by two faces and one of those faces displayed the same emotion as the target face. In this task, participants had to indicate which faces expressed the same emotion. Responses were given by pressing a button corresponding to the left or right emotion (emotion labeling) or left or right face (emotion matching), respectively. The visual stimuli (male and female faces of young adults) were obtained from the face database of the Max-Planck Institute (76). The five basic emotions expressed by these faces were happiness, sadness, anger, disgust, and fear. Following a fixation period of 250 ms, faces were presented focally on black background until a response was collected or 4,500 ms passed without a response. The order of the trials was randomized and different emotions appeared with equal probability. All participants completed 10 practice trials, followed by a test block with 40 trials for each task (emotion labeling and matching). For statistical analyses, reaction time on response-correct trials, and accuracy was extracted. Each variable was averaged across the two tasks and is now referred to as emotion recognition.

#### Statistical Analysis

Preliminary calculations: With a series of *t*-tests we explored, if shift working (yes vs. no) did systematically bias the pattern of results. Next, with a series of Pearson's correlations, we tested the associations between age, subjective symptoms of insomnia, perceived stress, mental toughness, and FER (speed; accuracy). With a series of *t*-tests, we tested gender differences in FER, age, mental toughness, perceived stress, and subjective symptoms of insomnia. Finally, we performed two separate multiple regression analyses for FER accuracy and speed to test whether and how subjective symptoms of insomnia are associated with FER after controlling for age, stress and mental toughness. The level of significance was set at alpha ≤ 0.05. The statistics were performed with SPSS® 25.0 (IBM Corporation, Armonk NY, USA) for Apple®.

## Results

### Preliminary Calculations

First, we explored, if working in shift (yes vs. no) did systematically bias the pattern of results. Compared to non-shift working police officers (*n* = 118), police officers working in shifts (*n* = 83) were younger [*M* = 34.03 years, (*SD* = 9.07) vs. 42.29 years (*SD* = 9.74); *t*_(199)_ = 5.98, *p* < 0.001, *d* = 0.88], they reported lower stress scores [*M* = 3.40 (*SD* = 2.22) vs. *M* = 5.06 (*SD* = 2.91); *t*_(199)_ = 4.19, *p* < 0.001, *d* = 0.64], and shift-working police officers reported higher Mental toughness scores [*M* = 3.74 (*SD* = 0.43) vs. *M* = 3.55 (*SD* = 0.42); *t*_(199)_ = 2.95, *p* < 0.01, *d* = 0.45]. For subjective symptoms of insomnia, and FER performance, means did not statistically significantly differ between shift working and non-shift working police officers (all Fs < 1; ps > 0.50).

### Main Calculations

Descriptive statistics of the sample and bivariate correlations between continuous study variables are reported in Table 1.

**Table 1:** Descriptive statistical indices and correlation coefficients between dimensions of facial emotion recognition and psychological dimensions.

	**Age**	**Perceived stress**	**Insomnia severity**	**Mental toughness**	**Facial emotion recognition**	
					**Reaction time**	**Accuracy**
	**M (SD)**	**M (SD)**	**M (SD)**	**M (SD)**	**M (SD)**	**M (SD)**
	38.55 (10.13)	4.28 (2.18)	7.83 (4.43)	3.62 (0.43)	1835.54 (282.76)	0.92 (0.06)
Age	–	0.00	0.00	0.05	0.17^*^	−0.13
Perceived stress	–	–	0.39^**^	−0.52^**^	−0.08	0.01
Insomnia severity	–	–	–	−0.34^**^	−0.05	−0.06
Mental toughness	–	–	–	–	0.06	−0.90
	–	–	–	–	–	–
**FACIAL EMOTION RECOGNITION**						
Reaction time	–	–	–	–	–	−0.40^**^
Accuracy	–	–	–	–	–	–

* *p < 0.05*;

** *p < 0.01*.

Performance on FER (accuracy; speed) was not associated with subjective symptoms of insomnia, nor with subjective stress or mental toughness. Poorer performance on FER (accuracy; speed) was associated with increased age.

Next, higher subjective symptoms of insomnia were associated with higher perceived stress and lower mental toughness scores.

For FER, age, subjective symptoms of insomnia, perceived stress, and mental toughness, no gender differences were observed (always trivial to small effect sizes; see Table 2).

**Table 2:** Descriptive and inferential statistical mean differences between male and female participants.

	**Sex**		**Statistics**
	**Male**	**Female**	
*N*	129	72	
	M (SD)	M (SD)	
Age	39.50 (10.52)	36.86 (9.22)	*t*_(199)_ = 1.77, *p* = 0.08, *d* = 0.27 [S]
Perceived stress	4.07 (2.55)	4.66 (3.13)	*t*_(188)_ = 0.40, *p* = 0.16, *d* = 0.21 [S]
Insomnia severity	7.61 (4.51)	8.24 (4.28)	*t*_(188)_ = 0.94, *p* = 0.35, *d* = 0.14 [T]
Mental toughness	3.68 (0.42)	3.52 (0.43)	*t*_(188)_ = 2.57, *p* = 0.01, *d* = 0.38 [S]
**FACIAL EMOTION RECOGNITION**			
Reaction time	1869.50 (297.65)	1774.32 (243.92)	*t*_(197)_ = 2.30, *p* = 0.02, *d* = 0.35 [S]
Accuracy	0.92 (0.07)	0.94 (0.04)	*t*_(197)_ = 2.91, *p* = 0.004, *d* = 0.35 [S]

*[T], trivial effect size; [S], small effect size*.

Finally, results from the multiple regression analyses confirmed the previous pattern of results: Shorter FER reaction time and higher accuracy was predicted by lower age, while levels of mental toughness, perceived stress, and subjective symptoms of insomnia did not reach statistical significance and were therefore excluded from the equation (see Tables 3A,B).

**Table 3A:** Multiple linear regression with facial emotion recognition reaction time as dependent variable, and age, perceived stress (PSS), insomnia severity (ISI), and mental toughness as predictors.

**Dimension**	**Variables**	**Coefficient**	**Standard error**	**Coefficient β**	***t***	***p***	***R***	***R*^**2**^**	**Durbin-Watson coefficient**
FER: reaction time	Intercept	1674.59	81.17	–	20.63	0.000	0.15	0.02	1.93
	Age	4.24	2.04	0.15	2.08	0.039	–	–	–
Excluded variables	Perceived stress	–	–	−0.08	−1.11	0.27	–	–	–
	Insomnia severity	–	–	−0.05	−0.71	0.48	–	–	–
	Mental toughness	–	–	0.05	0.69	0.49	–	–	–

*FER, facial emotion recognition*.

**Table 3B:** Multiple linear regression with facial emotion recognition accuracy as dependent variable, and age, perceived stress (PSS), insomnia severity (ISI), and mental toughness as predictors.

**Dimension**	**Variables**	**Coefficient**	**Standard error**	**Coefficient β**	***t***	***p***	***R***	***R*^**2**^**	**Durbin-watson coefficient**
FER: accuracy	Intercept	0.95	0.02	–	51.46	0.000	0.12	0.015	1.76
	Age	−0.001	0.000	−0.12	−2.67	0.04	–	–	–
Excluded variables	Perceived stress	−0.001	−0.002	−0.02	−0.25	0.80	–	–	–
	Insomnia severity	−0.001	−0.001	−0.09	−1.18	0.24	–	–	–
	Mental toughness	−0.02	−0.013	−0.13	−1.49	0.14	–	–	–

*FER, facial emotion recognition*.

## Discussion

The key findings of the present study are that among a larger and broad sample of adults, performance on FER was unrelated to subjective symptoms of insomnia. Moreover, no systematic gender differences appeared in FER, subjective symptoms of insomnia, perceived stress, and mental toughness. The present pattern of results adds to the current literature in an important way: it appears that FER is unrelated to subjective symptoms of insomnia, understood as a proxy of quality of sleep.

Two research questions were formulated, and each of these will now be discussed.

With the first research question, we examined the association between FER and subjective symptoms of insomnia, and data showed a zero-association. This pattern of results is in accord with a wealth of studies showing a very similar pattern (43–45), and accordingly, the present results are in contrast to those studies showing that sleep (in terms of shorter sleep duration) and lower performance in FER were associated (9, 38–42).

With the second research question, we examined whether FER would be more impaired among females, compared to their male counterparts, but data did not support this assumption. Accordingly, the present pattern of results did not match the observations of van der Helm et al. (42).

The quality of the data does not allow a deeper introspection into the underlying psychological mechanisms to explain the zero-association between FER and subjective symptoms of insomnia.

However, we observed that increasing age and lower performance on FER were associated. Such associations are well-known from cognitive tests, with lower test performances observed in people or more advanced age (77, 78). Likewise, Mograss et al. (39) and Sheth et al. (46) tested long-term memory performance for faces, and one might question whether and to what extent memory processes of encoding, storage and retrieval of faces could be understood as emotional or cognitive processes [see (79, 80) for overview of memory processes].

Furthermore, the study set up was designed to be ecologically valid; this is to say, participants reported their subjective symptoms of insomnia reflecting their real-life conditions. By contrast, the majority of studies reporting changes in FER manipulated sleep duration experimentally (38, 39, 41–43, 49, 50), thus putting participants under artificial conditions of sleep deprivation. In this view, one might question the ecological validity of sleep deprivations lasting for 47 h (49) or even 72 h (41). Next, Van Dongen et al. (22) showed in their studies on the dose-response of sleep deprivation and cognitive performance that a total sleep deprivation of 72 h led to a sharp and dramatic decrease of cognitive alertness and working memory performance. In other words, we observed that sleep deprivation leads to a deterioration of cognitive performance, while FER remained fairly stable, even if we take into account those studies showing a decreased performance in FER; specifically, after a total sleep deprivation of 47 h (49) or 72 h (41), one might expect an even sharper decrease in FER performance.

While, again, the quality of the data does not allow a deeper understanding of the underlying neurophysiological processes of the present pattern of results, a number of studies provide possible explanations. Yoo et al. (81) showed with their imaging studies with adults with insomnia and healthy controls, that under sleep restriction the amygdala displayed dramatically increased neuronal activation and response to negative emotional stimuli. Such a process is believed to be associated with a decrease in top-down, prefrontal control. Further, Yoo and colleagues speculated that a night of sleep appears to “reset” the correct brain reactivity to next-day emotional challenges. Such a “reset” should enable the maintenance of functional integrity of the amygdala circuit. In this vein, Heberlein et al. (82) showed that ventromedial and orbitofrontal regions of the PFC appeared to be crucial for FER. However, processes of FER do not appear to exclusively undergo such prefrontal loops. Rather, it is claimed that further brain regions such as the amygdala, fusiform, superior colliculus, insular cortex, and hippocampus are involved in such processes (9, 40). Thus, while Motomura et al. (40) claimed that the subcortical and temporoparietal areas are important for FER and that these regions are sensitive to sleep loss, further imaging and metabolic studies might investigate why, despite the sensitivity to sleep loss of such regions, performance on FER remains broadly unaffected, if compared to dramatically impaired cognitive processes (22).

Next, we know from studies on the cognitive performance of patients with Restless Legs Syndrome (RLS) that, despite their seriously impaired sleep quality, their performance on cognitive tests was not necessarily lower and could even be higher (83) than that of healthy controls (84–86), [but for contrary findings see, for instance, Fulda et al. (87, 88)]. To explain the lack of poorer cognitive performance among patients with RLS, Gamaldo et al. (83) speculated that these patients might have undergone a process of sleep loss adaptation. That is to say, despite their relative loss of sleep, they are able to produce proficient cognitive performances when necessary.

Last, from an evolutionary point of view, it is important to quickly identify faces and to distinguishing between others' different mood states and possible emotions (3, 6, 7). Accordingly, it is conceivable that such evolutionarily well-rooted detection systems evolved to keep social interaction and social relationships stable and unaffected from current quality and duration of sleep.

Despite the novelty of the study results, several limitations warrant against overgeneralizations. First, we did fully rely on self-reports, which by definition could be biased. Accordingly, in future studies psychiatrists and clinical psychologists might assess participants' sleep and psychiatric status more thoroughly. Second, to assess the quality of sleep, we used the Insomnia Severity Index (65), a self-rating scale for insomnia. While, thus, strictly taken, participants did not report, if they were suffering for instance from sleep apnea, snoring, RLS or hypersomnia, the Insomnia Severity Index is an excellent tool to assess sleep quality (or the lack of sleep quality), regardless of an underlying sleep-disordered breathing, RLS or period limb movements. Accordingly, we hold that the tool allowed participants to thoroughly assess and report their quality of sleep. Third, FER was tested under static, but not dynamic conditions. This might be considered a limitation, as static conditions might be rare, while dynamic conditions might reflect real life conditions more thoroughly. Accordingly, future studies should consider to assess FER performance of dynamic faces. Fourth, the sample consisted of police officers; accordingly, it is conceivable that the sample was both physically and mentally particularly healthy, as police officers regularly undergo medical checks and briefings of their mental health status. Nevertheless, we considered police officers to be an especially interesting population because they are often exposed to situations where they have to interpret other people's emotions and non-verbal behavior to find out whether they are honest or lying to them. Furthermore, interpreting emotions swiftly and correctly might be particularly helpful in policing to predict behavior in extreme situations that possibly involve physical violence or even existential threats. Thus, efficient FER is expected to facilitate the fulfillment of professional duties. Likewise, and perhaps unlike other professionals, police officers can benefit from internal sports and health courses and psychological advice. Fifth, the participation in the study was completely voluntary. Accordingly, only participants able and willing to comply with the study conditions took part, which may have entailed a recruitment and selection bias (in the sense of a “healthy worker effect”). Sixth, it is also possible that latent and unassessed psychological and physiological factors might have biased two or more dimensions in the same or opposite directions.

## Conclusions

In a broad occupational sample of adults, subjective symptoms of insomnia and performance in a FER test were unrelated. Thus, FER appears to be resistant to subjective symptoms of insomnia. Further studies might replicate this pattern of results with other participants such as adolescents and older adults, and healthy and clinical samples.

## Author Contributions

SB, RS, SL, FC, DS, EH-T, UP, and MG: study design, interpretation of the data; RS, SL, FC, and MG: data gathering; SB, RS, SL, DS, and MG: writing the draft; SB, RS, SL, FC, DS, and MG: integration of the authors' comments; SB, RS, SL, FC, DS, EH-T, UP, and MG: final manuscript.

### Conflict of Interest Statement

The authors declare that the research was conducted in the absence of any commercial or financial relationships that could be construed as a potential conflict of interest.
